# Intraoperative Difficulties and Postoperative Complications Associated With Cochlear Implantations: A Study From Erbil City

**DOI:** 10.7759/cureus.52106

**Published:** 2024-01-11

**Authors:** Lana Shahabaddin, Said Al-Jaaf, Abdulkhaliq Emin

**Affiliations:** 1 Otolaryngology - Head and Neck Surgery, Hawler Medical University, Erbil, IRQ; 2 Otolaryngology, Hawler Medical University, Erbil, IRQ

**Keywords:** postoperative wounds, complications’, surgical wound infection, cerebrospinal fluid (csf), cochlear implant (ci)

## Abstract

Background and objective

Cochlear implants are highly effective for the treatment of severe to profound hearing loss. Cochlear implant surgery is a safe surgical procedure; however, due to many modifications over the years, it has been associated with certain minor and major complications. This study aimed to examine the intraoperative difficulties and postoperative complications in patients who received cochlear implants in Erbil City.

Methods

We conducted a retrospective descriptive study regarding complications of cochlear implants involving patients who received unilateral or bilateral cochlear Implants at the Rizgary Teaching Hospital and a private hospital in Erbil City from January 2013 to July 2022. Their medical records were analyzed, and data on demographics, intraoperative difficulties, and postoperative complications were gathered.

Results

A total of 160 patients with cochlear implants (87 male, 54.4%; 73 female, 45.6%) were included in the study. The mean age of the patients at the time of operation was 6.76 ± 8.02 years (range: 1-53 years); 150 of these patients were children and 10 were adults (18 years and above). Intraoperative difficulties occurred in five patients (3.1%). The overall rate of complication was 10%, 7.5% of which was minor (the most common being wound infection) and 2.5% major (the most frequent being device extrusion).

Conclusions

Cochlear implant surgery is a safe procedure performed to treat profoundly deaf patients. It is associated with a low rate of complications, most of which can be successfully managed with conservative measures or minimal intervention.

## Introduction

Sensorineural hearing loss is most commonly caused by deformity or damage to the cochlear structure or auditory nerve, which is difficult to correct by medical or surgical intervention [1]. Cochlear implantation (CI) is the best surgical option for effectively managing bilateral severe to profound sensorineural hearing loss in both children and adults, and these implants are equivalent to conventional hearing aids in their function and efficacy [2]. CIs are different from conventional hearing aids as they bypass the injured peripheral sensory structures, transmitting impulses to the spiral ganglion, which in turn transmits them to the cranial nerve VIII [3]. The CI technique is regarded as safe and associated with a low rate of complications [4]. With the demand for CI consistently on the rise globally in the last few decades, patients, their relatives, and surgeons should be aware of the post-CI complications and the management of these complications [5].

Post-CI complications involving various devices have been well-documented in studies conducted in different parts of the world [6,7]. The first report of postoperative complications in patients undergoing CI was published in 1995 by Cohen et al., who also classified these complications into two types: major and minor [8]. The reported intraoperative CI complications include damage to surrounding structures (sigmoid sinus, chorda tympani, and facial nerve), difficulty with the insertion of implants, and cerebrospinal fluid (CSF) leak. The postoperative complications following cochlear implant surgery could be classified according to their severity and timing. Major postoperative complications are defined as those that occur during or after cochlear implant surgery and require a major surgical intervention or those requiring the removal of the device. These include facial nerve paralysis, device failure, flap necrosis, electrode migration, magnet displacement, perforated eardrums, meningitis, cholesteatoma, or other dangerous complications causing persistent distress or dysfunction or resulting in a permanent disability. The minor complications are those that could be managed with medical treatment or minor surgery and include wound seroma or hematoma, acute middle ear inflammation, transient vertigo, wound infection, transient facial palsy, or chorda tympani nerve damage [9,10].

The rate of post-CI complications varies among studies in the literature, ranging from 6 to 36% [11]. Technological advances over the years have led to a significant decrease in the rates of postoperative complications following CI, and these methods have primarily focused on preventing device failure and pertain to improved surgical methods such as fixation techniques, alongside minimal incisions [11,12]. Cochlear implant surgery has gained immense popularity in recent years thanks to the advances in technology but certain congenital, financial, and logistical obstacles remain [13]. There is scarce data on the outcomes and complications following cochlear implant surgery in the literature. In light of this, this study aimed to retrospectively review the adverse effects and difficulties associated with cochlear implant surgery, including minor and major postoperative complications, as well as discuss the management and prevention of such complications.

## Materials and methods

A retrospective descriptive study was conducted involving patients who received unilateral or bilateral CI at the Rizgary Teaching Hospital and a private hospital in Erbil City, Kurdistan region, Iraq from January 1, 2013, to July 30, 2022. The medical records of 162 patients were reviewed, and data on demographics, as well as intraoperative difficulties and postoperative complications after CI were gathered. The inclusion criteria were as follows: patients with a history of bilateral profound or severe-to-profound sensorineural hearing loss and who did not benefit from binaural amplification. Patients with inner ear anomalies and two patients with ossifications of the cochlea post-meningitis were excluded, as implants could not be inserted in them.

The diagnosis involved an audiological evaluation [aided audiometry, auditory brainstem response (ABR), and otoacoustic emission]. The preoperative radiologic findings were obtained from both MRI and high-resolution CT (HRCT) scans of the temporal bone that were acquired via slices with a thickness of <1 mm.

A total of 162 implants were performed during the study period; the types of implants used were as follows: Nucleus manufactured by Cochlear Corp., Lane Cove, Australia (n=153) and Advanced Bionics manufactured by Advanced Bionics, Sylmar, CA (n=9); 83 of the surgeries were done at the Rizgari Teaching Hospital by a team of experienced surgeons and 76 operations were done at a private hospital by a single experienced surgeon.

Cochlear implant surgeries

The surgeries were conducted in a highly sterile environment, with facial nerve monitoring conducted throughout the operation. Prophylactic antibiotics were given intraoperatively. A lazy S postauricular incision, with a limited subperiosteal pocket for the device base, was used. A bony bed was drilled and prepared for the receiver-stimulator. A cortical mastoidectomy was performed, the short process of the incus was visualized, and a posterior tympanotomy was done and enlarged to approach the round‐window niche. In some cases, finding the location of the round window was not easy, and cochleostomy was performed in those cases; 1 ml of Decadron was injected into the round window. After electrode insertion, the round window and the posterior tympanotomy were sealed with soft tissue; auditory nerve response telemetry and/or neural‐response telemetry were performed.

The study protocol was approved by the Ethics Review Board of Hawler Medical University. Statistical analysis was performed using SPSS Statistics, version 24 (IBM Corp., Armonk, NY).

## Results

A total of 160 patients with cochlear implants (87 male, 54.4%; 73 female, 45.6%) were included in the study. The mean age of the patients at the time of operation was 6.76 ± 8.02 years (range: 1-53 years); 150 of these patients were children and 10 were adults (18 years and above). The types of hearing loss in the cohort were as follows - prelingual: n=135 (84.4%), perilingual n=10 (6.3%), and postlingual: n=15 (9.4%). There was a family history of hearing loss in 103 (64.4%) patients: first-degree consanguineous (third-degree relative) marriage was reported in the families of 64 (40%) patients and with a fourth-degree relative in the families of 47 (29.4%) patients; such instances were not reported in the families of 49 (30.6%) patients.

Among the children, 81 were male (54%) and 69 female (46%). The types of hearing loss in children were as follows - prelingual: n=132 (88%), perilingual: n=9 (6%), and postlingual: n=9 (6%) patients. As for adults, six (60%) were male and four female (40%). The types of hearing loss in adults were as follows - prelingual: n=3 (30%), perilingual: n=1 (10%), and postlingual: n=6 (60%). Regarding the side of CI, 153 (95.6%) were right-sided implants, four (2.5%) were left-sided, and three were bilateral implantation (sequential) (1.9%). Intraoperative difficulties occurred in five patients (3.1%).

Three patients had insertion-related difficulties (two children and one adult); all of them had a history of meningitis. One child had a CSF gusher; there was a wide internal auditory canal on MRI, but no other inner anomalies were found by imaging. During surgery, when the cochleostomy was done, the CSF gusher occurred. Once its flow decreased, controlled hyperventilation was initiated and intravenous propofol and mannitol were administered; elevation of the head was done with good sealing of the cochleostomy by soft tissue after the insertion of the device, and the patient did not develop complications following the surgery (Table 1).

**Table 1:** Intraoperative difficulties among studied patients CSF: cerebrospinal fluid

Intraoperative difficulties		
	Frequency	Percent
CSF gusher	1	0.6
Sigmoid sinus injury	1	0.6
Insertion difficulties	3	1.9
None	155	96.9
Total	160	100

A 30-month-old child had a sigmoid sinus injury; the sigmoid sinus was superficial and anteriorly located from the start of drilling, and the sigmoid sinus was injured with severe bleeding. It was controlled by Surgicel and Gelfoam pack. The overall rate of complication was 10%, 7.5% of which was minor (the most common being wound infection) and 2.5% major (the most frequent being device extrusion) (Tables 2, 3). Minor complications occurred in 12 patients, all of whom were children except for a patient with transient facial palsy.

**Table 2:** Minor postoperative complications among studied patients

Minor postoperative complications		
	Frequency	Percent
Nausea and vomiting	2	1.3
Transient facial palsy	1	0.6
Wound infection	7	4.4
Hematoma	1	0.6
Acute otitis media	1	0.6
None	148	92.5
Total	160	100

**Table 3:** Major postoperative complications among studied patients

Major postoperative complications		
	Frequency	Percent
Seroma	1	0.6
Device extrusion	2	1.3
Device failure	1	0.6
Magnet Displacement	1	0.6
None	155	96.9
Total	160	100

**Figure 1:**
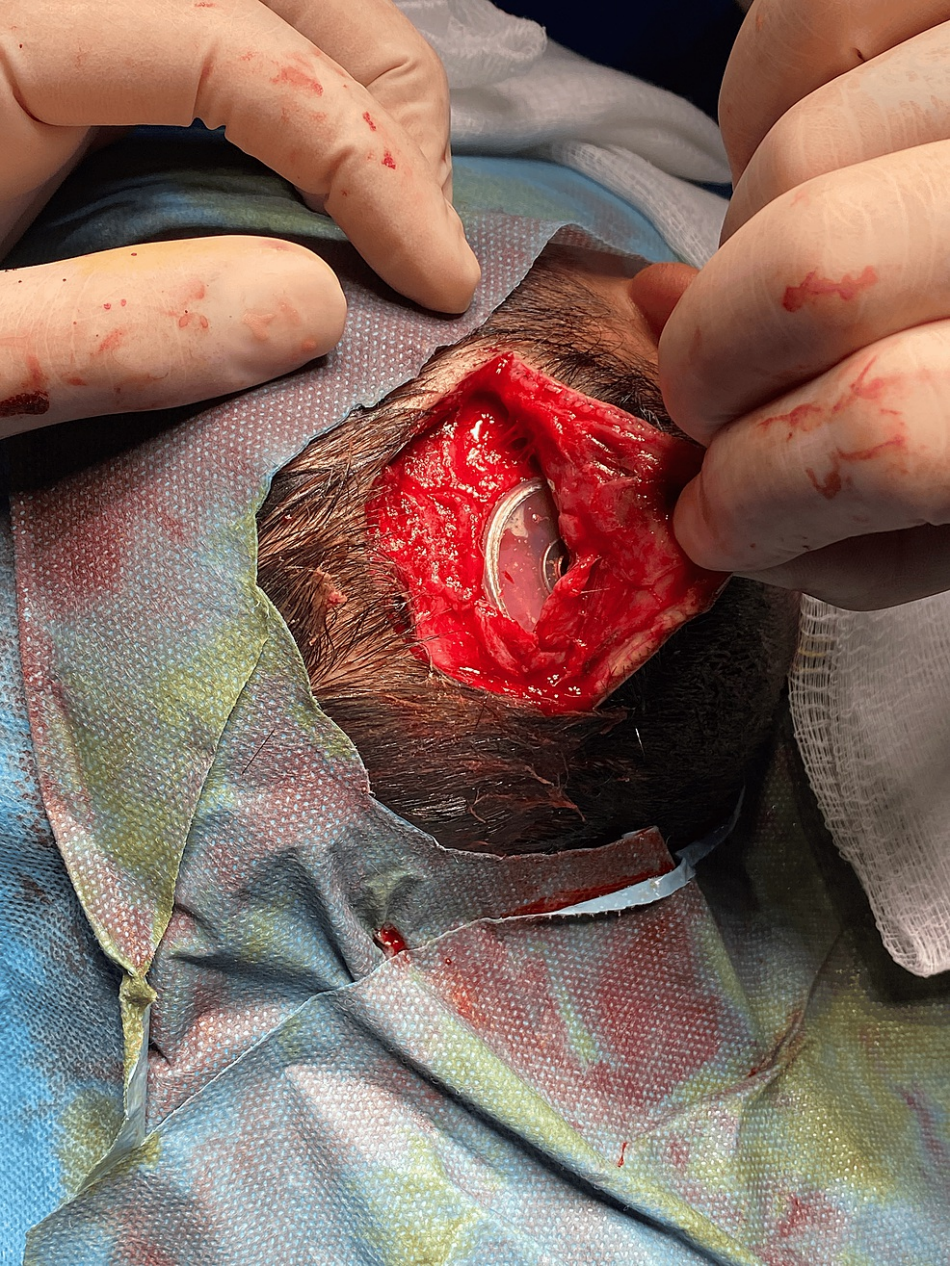
Magnet migration

One of the pediatric patients had magnet migration following trauma (falling). Reposition of the magnet was done with an operation. Another pediatric patient experienced device extrusion due to the anteriorly located device pressing on the skin (Figure 1). Facial palsy was reported in a 23-year-old male patient two days postoperatively, which was treated with systemic antivirals and steroids, and the patient had a full recovery.

**Figure 2:**
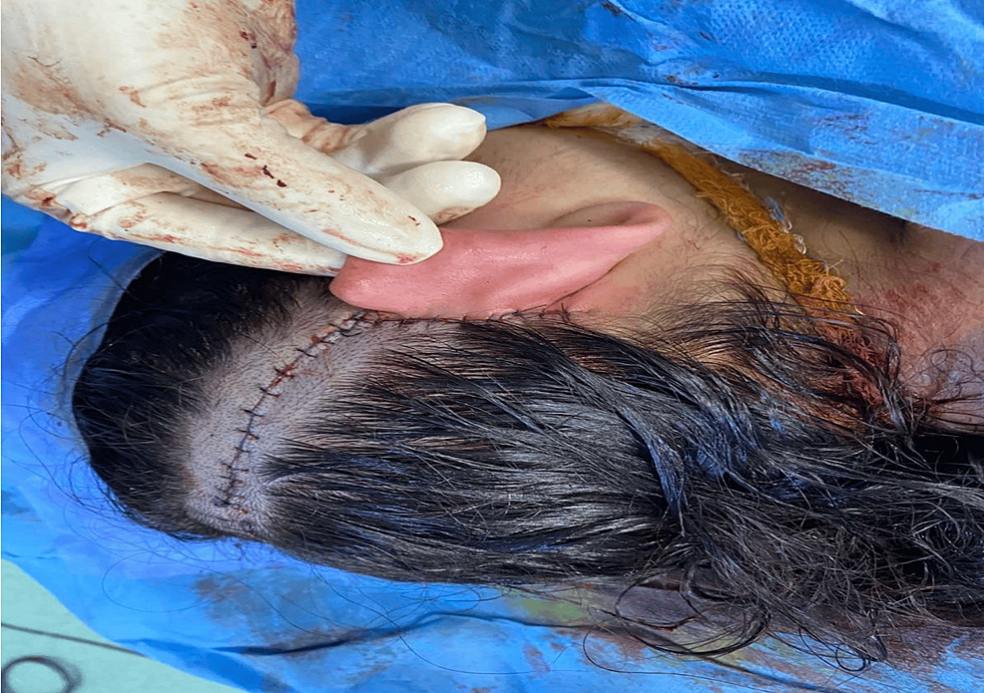
Rotational flap

Two children had nausea and vomiting postoperatively, which was controlled with medications. Seven children had simple wound infections and were treated by application of local antibiotics and temporarily removing the device for a few days. A seven-year-old child developed a hematoma one week postoperatively, which was managed by aspiration of the hematoma, with no sequelae. Another child had acute otitis media, and he received empiric oral antibiotic treatment with complete improvement. Major complications occurred in five patients, and four of them were children. One child developed a seroma two years following implantation with thinning of the skin over the device, which was treated by aspiration of the seroma, and a rotational flap was done (Figure 2).

**Figure 3:**
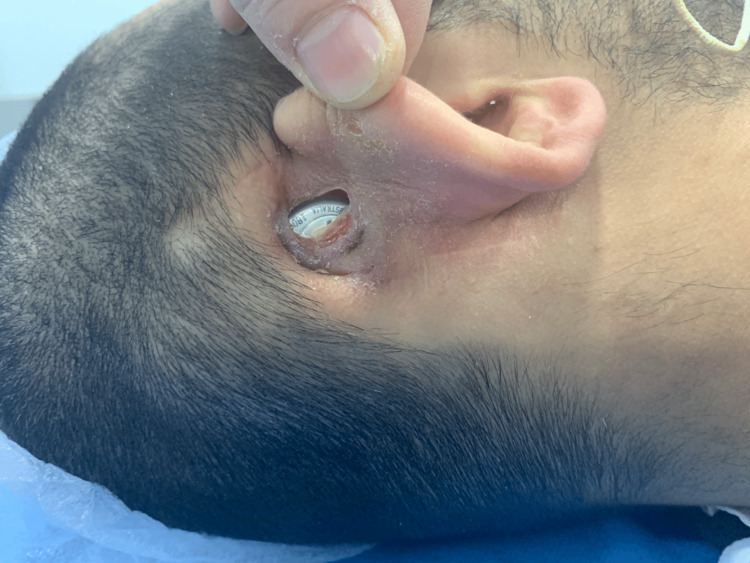
Device extrusion

**Figure 4:**
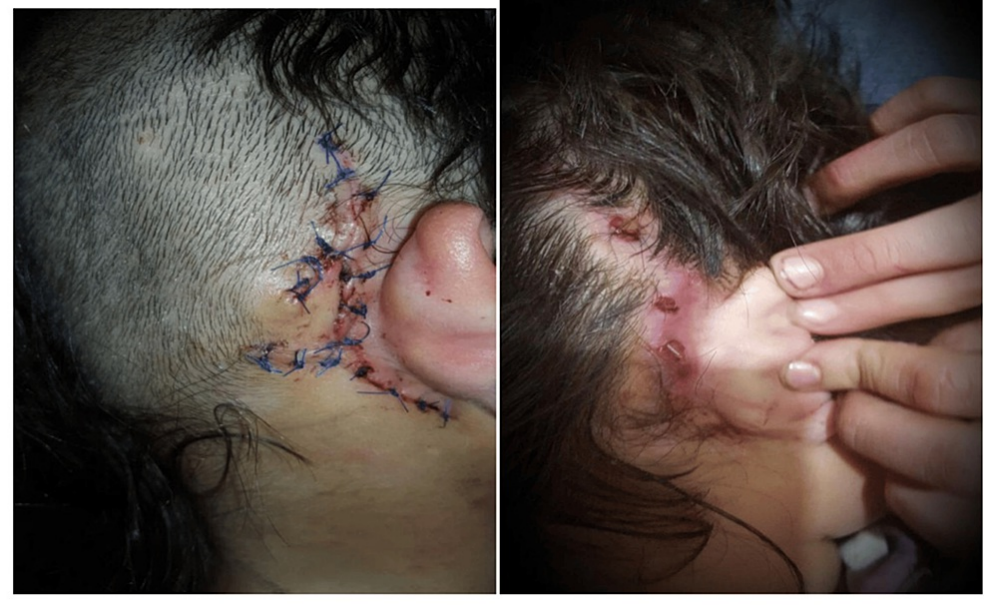
Flap necrosis

A 23-year-old patient developed a severe skin infection, which was neglected and led to device extrusion (Figure 3). A child developed a skin infection, and flap necrosis (right-side implant), which was managed initially by a rotational flap; however, eventually the flap failed with device extrusion, and a re-implant was performed on the left side (Figure 4). One child developed hard device failure (Advanced Bionics); he also developed chronic suppurative otitis media, which has failed to respond to medical treatment.

## Discussion

Complications of CI, although not common, may cause morbidity and place extra financial burdens on the patients and families involved. In our study, the overall complication rate was 10%: 7.5% minor and 2.5% major. The most common minor complication was wound infection and the most common major complication was device extrusion, which aligns with the results of other studies in the literature. In a study by Awad et al. [14], the overall complication rate was 10.43%: 3.68% major and 6.75% minor. The most common minor complication was wound-related complications, and the most common major complication was device failure. Besides device failures, the other major complication rates stood at 1.84% [14]. A study by Halawani et al. [15] reported an overall complication rate of 10.2% (n=105), of which 98 cases (9.5%) had minor complications and seven cases (0.7%) had major complications. The most common complications were seroma/hematoma (n=22, 21%) followed by pain (13.2%), wound infections (11.4%), and vertigo (9.5%) [15]. In the review by Shiras et al. [16], the mean age of implantation was 3.03 years (range: 1-4.11 years). The overall complication rate was 7.51%, which comprised both major (2.34%) and minor (5.16%) complications.

The most common major complications were flap-related issues and the most frequent minor complication was facial paralysis [16]. In a study by Garrada et al. [17], complications occurred in 28 (19%) patients, including minor complications in 17 (11.5%) and major complications in 11 (7.4%). A study by Ozdemir et al. [18] documented an overall complication rate of 13.68%, involving 8.28% minor and 5.4% major complications. Swelling (wound seroma or hematoma) was the most common minor complication. The most common cause of major complications was related to implanted devices [18]. In A study involving 1452 CI procedures, the rate of major complications in 19 years was 4.75% (69 patients) and the rate of minor complications was 5.4% (79 patients). The overall complication rate was 10.1% (148 patients) [19].

In a study by Binnetoglu et al. [20], the overall rate of complications was 3.7% (n=97), including 78 cases (3.0%) that developed minor complications and 19 cases (0.7%) that developed major complications. The most commonly occurring minor complication in our study was vertigo, and the most commonly occurring major one was implant extrusion [20]. We believe that this broad range in the rate of complication may be due to the variations in the number of patients included, the classification of complications, and the different surgical techniques employed. Our study included 160 patients treated in a period spanning nine years and the cochlear implant surgery is not covered by insurance in our locality.

Regarding intraoperative difficulties in our study, three patients had insertion difficulties (two children and one adult), and all of them had a history of meningitis. Cochleostomy was done in all of them to ensure proper insertion of the electrodes; none of these three patients experienced postoperative complications. One child had a CSF gusher (patients with congenital ear anomalies were excluded from the study). The gusher was stopped without further sequelae, which is in line with other studies that found that intraoperative CSF gusher occurs in various types of inner ear malformations but could occur less frequently in cases without radiological abnormalities and can be treated without further complications [21,22]. A 30-month-old child had a sigmoid sinus injury; the sigmoid sinus was superficial and anteriorly located from the start of the drilling. The sigmoid sinus was injured with severe bleeding and it was controlled by Surgicel and Gelfoam pack. In a study by Ma et al. [23] involving 538 CI children, four cases had significant sigmoid sinus anterior displacement, and injury to the sigmoid sinus was avoided by the removal of the incus and abrasive reduction of the posterior wall of the bony external acoustic meatus.

Minor complications occurred in 12 patients, all of whom were children except for a patient with transient facial palsy. Major complications were also more common in the pediatric group (n=4 plus one adult patient), which is similar to the findings of a study by Al Shaikh et al. [24]. However, our results in this regard contrast with those of Farinetti et al, who reported that the complication rate was significantly higher in the adult population [25]. Transient facial palsy was observed in a 23-year-old male two days postoperatively, and he had a full recovery following medical treatment. The delayed onset of facial nerve palsy can be attributed to many causes, such as an inflammation process of the nerve or the surrounding tissue after wound healing [26], as well as direct pressure on the nerve, caused, for instance, by the cochlear implant electrode, an infection, or reactivation of herpes viruses [27]. The literature review revealed that most cases of delayed-onset facial nerve paresis following CI occur within the first month of implantation, and the prognosis for recovery is promising for these patients [28]. Seven children had simple wound infections, which were treated medically. One child developed a hematoma and was treated by aspiration; two children who developed severe nausea and vomiting on the first day postoperatively responded well to medical treatment. One child developed acute otitis media a few weeks following implantation, which resolved with medical treatment.

As for major complications, one child developed a seroma two years following implantation with thinning of the skin over the device, which necessitated the aspiration of the seroma, and a rotational flap was done. Qin et al. have reported that seroma may be simple and treated by aspiration, pressure dressing, and antibiotics; in other patients, the skin over the implant ruptured, leading to implant extrusion [29]. In our study, one child developed magnet migration following trauma, which was managed surgically by repositioning the magnet. Wild et al. have also discussed magnet migration; it involves the implanted magnet migrating out of its central location within the internal receiver-stimulator aerial pocket. Several such cases have been described in the literature, mostly following trauma [30]. Alahmadi et al. [31] have stated that once the diagnosis of magnet dislocation has been established, most cases would require surgical repositioning or replacement of the magnet; a systematic review revealed that MRI and head trauma were the most common causative factors for magnet displacement.

Two instances of device extrusion were observed in our study, and one of them (an adult patient) was due to a neglected infection four years following the implantation; the device was removed surgically, and re-implantation was not done as per the request of the patient and his family. A child developed a skin infection, and flap necrosis, which was managed initially by a rotational flap but failed, and device extrusion happened because of an anteriorly located device pressing on the skin leading to infection. A new implant was inserted in the left ear. To avoid this complication, the device is positioned more backward and upwards, and placing it on a straight plateau enables the device to void the pressure from the edge of the device on the skin. A 23-year-old patient developed a severe skin infection five years following the implantation, which was neglected and hence led to device extrusion. The device was removed surgically and the patient and his family refused re-implantation.

Bi et al. [2] have described patients in whom revision surgery failed with severe flap rupture and implant device extrusion, and the removal of the device ultimately led to wound healing in all cases. Implant removal and contralateral or ipsilateral cochlear implant should be considered in such cases. A study by Singh et al. [32] reported two cases with flap necrosis, and flap rotation was done in both these cases with antibiotic coverage and dressings, and the implant was salvaged. One child developed internal device failure (Advanced Bionics) approximately eight years after implantation in the right ear; he also developed right chronic suppurative otitis media, which has not responded to medical treatment. The patient is scheduled to have his implant removed. In a study by Chen et al. [33], device failure was the most common reason for revision surgery, followed by infection.

## Conclusions

CI is a safe surgical procedure that is effective for treating profoundly deaf patients. It is associated with a low rate of complications, and most of them can be successfully managed with conservative measures or minimal intervention. Regardless, both physicians and patients must be aware of the potential adverse effects and risks posed by cochlear implants, especially since these patients require lifelong follow-up.
